# Selection of Reference Genes for RT-qPCR Analysis Under Extrinsic Conditions in the Hawthorn Spider Mite, *Amphitetranychus viennensis*

**DOI:** 10.3389/fphys.2020.00378

**Published:** 2020-04-21

**Authors:** Jing Yang, Yuying Zhang, Jin Zhao, Yue Gao, Zhongfang Liu, Pengjiu Zhang, Jianbin Fan, Xuguo Zhou, Renjun Fan

**Affiliations:** ^1^College of Plant Protection, Shanxi Agricultural University (Institute of Plant Protection, Shanxi Academy of Agricultural Science), Shanxi Key Laboratory of Integrated Pest Management in Agriculture, Taiyuan, China; ^2^Department of Entomology, University of Kentucky, Lexington, KY, United States; ^3^Research Institute of Applied Biology, Shanxi University, Taiyuan, China

**Keywords:** RT-qPCR, reference gene, extrinsic conditions, dietary RNAi, hawthorn spider mites, *Amphitetranychus viennensis*

## Abstract

Hawthorn spider mite, *Amphitetranychus viennensis* Zacher, is an economically important arthropod pest for fruit trees and woody ornamental plants. Extensive and repetitive use of synthetic acaricides has led to the development of resistance in *A. viennensis*. To understand the molecular basis of pesticide resistance, and to develop genetic-based control alternatives (e.g., RNAi-based biopesticides), a standardized protocol for real-time quantitative reverse transcription PCR (RT-qPCR) is needed. In the proceeding phase of this research, we screened for the internal references for RT-qPCR analysis from a pool of *A. viennensis* housekeeping genes under the intrinsic conditions, including developmental stage, sex, and diapause. Here, we continued our efforts to search for the reference genes under an array of extrinsic conditions, including temperature, humidity, photoperiod, host plant, and dietary RNAi. The stability of these candidate reference genes was investigated using geNorm, NormFinder, BestKeeper, and ΔCt method, respectively. Finally, RefFinder, a statistical platform integrating all four algorisms, provided a comprehensive list of genes for each extrinsic condition: (1) *EF1A*, α*-tubulin* and *Actin3* were the best candidates for temperature, (2) *GAPDH*, *18S*, and *Actin3* were the most stable genes for humidity, (3) *V-ATPase B*, *Actin3*, and *18S* were the top reference genes for photoperiod, (4) *GAPDH*, *V-ATPase B*, and α*-tubulin* were recommended for host plants, and (5) *GAPDH*, *V-ATPase B*, and *RPS9* were the top choices for dietary RNAi. Overall, *V-ATPase B*, *GAPDH*, and *Actin3* were the most commonly selected reference genes in *A. viennensis* regardless of the experimental conditions, including both intrinsic and extrinsic. Information present here lays the foundation for the genomic and functional genomic research in *A. viennensis*.

## Introduction

A better understanding of gene expression profiles can provide molecular insight into complex mechanisms underlying the effects of stressors on organisms (Nolan et al., 2006). Real-time quantitative reverse transcription PCR (RT-qPCR) is one of the most effective methods for quantifying gene expression levels because of its specificity, reproducibility, and sensitivity (Ginzinger, 2002; Derveaux et al., 2010). The accuracy of this technique, however, can be affected by PCR amplification, primer performance and sample quantity (Bustin, 2002; Bustin et al., 2005; Strube et al., 2008). Therefore, reliable endogenous reference genes should be used to eliminate bias and reduce errors (Vandesompele et al., 2002). Ideally, the stability of reference genes should be independent of experimental conditions. Housekeeping genes (HKGs), which are involved in basic metabolism and cellular maintenance, have been used extensively as reference genes for RT-qPCR analysis (Lü et al., 2018).

The hawthorn spider mite, *Amphitetranychus viennensis* Zacher, is an economically important cell-content feeding pest (Bensoussan et al., 2016, 2018) for fruit trees and woody ornamental plants in the Palaearctic region (Ehara and Gotoh, 1990). It can concurrently infest various host plants within Rosaceae family (Ehara and Gotoh, 1990). Traditionally, *A. viennensis* management has been relied heavily on synthetic chemicals, i.e., insecticides and acaricides. As a result, *A. viennensis* has developed resistance to a wide range of commercially available acaricides (Li et al., 2007). The pesticide resistance, combined with residue issues in both food products and the environment, has led to the search for control alternatives for *A. viennensis*.

The advent of genomics era has provided an unprecedented opportunity to overcome this barrier and explore new ways to managing pest problems in a long-term and sustainable fashion (Xu et al., 2015). RNA interference (RNAi), a common post-transcriptional gene silencing mechanism in eukaryotic organisms, including insects and mites, has recently been developed as an alternative strategy to control agricultural pests (Baum et al., 2007; Gordon and Waterhouse, 2007; Price and Gatehouse, 2008; Kwon et al., 2013; Xu et al., 2015; Suzuki et al., 2017; Yoon et al., 2018). With the deregulation of an RNAi trait by the United States regulatory agencies, the addition/commercialization of this biotechnology into the pest control arsenal is a matter of time. The lack of genomic resources in non-model systems, such as *A. viennensis*, has become a limiting factor for such efforts.

To facilitate the development of new pest management strategies, specifically RNAi, in *A. viennensis*, we have established genomic resources (unpublished data). To take advantage of these newly sequenced genome and transcriptomes, a standardized RT-qPCR procedure following the MIQE (Minimum Information for publication of Quantitative Real-Time PCR Experiments) guidelines (Bustin et al., 2009) is warranted for the subsequent genomic and functional-genomic research in *A. viennensis*.

Recently, selection of reference genes has been conducted for an array of cell-content feeding arthropods, including three spider mites species, *Tetranychus urticae* (Yang et al., 2015a), *Tetranychus cinnabarinu* (Sun et al., 2010), and *Panonychus citri* (Niu et al., 2012), and many Hemiptera species, *Myzus persicae* (Kang et al., 2017), *Aphis gossypii* (Ma et al., 2016), *Toxoptera citricida* (Shang et al., 2015), *Lipaphis erysimi* (Koramutla et al., 2016), *Sogatella furcifera* (An et al., 2016), *Aphis craccivora* (Yang et al., 2015b), *Bemisia tabaci* (Li et al., 2013), *Phenacoccus solenopsis* (Arya et al., 2017), *Nilaparvata lugens* (Yuan et al., 2014), *Acyrthosiphon pisum* (Yang et al., 2014), and *Ericerus pela* (Yu et al., 2016). In addition, we previously screened for the internal references under intrinsic conditions from a pool of *A. viennensis* HKGs (Yang et al., 2019).

In this study, we continued our efforts to search for the reference genes under an array of extrinsic conditions, including temperature, humidity, photoperiod, host plant, and dietary RNAi. The stability of these nine candidates, including *18S ribosomal RNA* (*18S*), *28S ribosomal RNA* (*28S*), *Elongation factor 1-alpha* (*EF1A*), β*-actin*, *Actin3*, *V- type proton ATPase subunit B* (*V-ATPase B*), α*-tubulin*, *40S ribosomal protein S9* (*RPS9*) and *Glyceraldehyde 3-phosphate dehydrogenase* (*GAPDH*), was investigated using geNorm, NormFinder, BestKeeper, and ΔCt method, respectively. Finally, RefFinder, a statistical platform integrating all four above-mentioned algorisms, provided a comprehensive list of reference genes for each extrinsic condition. This study is the first step toward establishing a standardized qRT-PCR analysis protocol for this agriculturally important cell-content feeding arthropod.

## Materials and Methods

### *Amphitetranychus viennensis* Colony Maintenance

*Amphitetranychus viennensis* (Acari: Tetranychidae) colonies were collected from crabapple *Malus* “Radiant,” at Shandong Agricultural University, Tai’an, Shandong province (36° 11′ 47^″^ N, 117° 07′ 10^″^ E), in August, 2017. *A. viennensis* larvae and adults were maintained in growth chamber at 26 ± 0.5°C, 16L: 8D photoperiod and 50% relative humidity (RH), and provisioned with fresh peach, *Prunus davidiana*, leaves.

### Total RNA Extraction and cDNA Synthesis

*Amphitetranychus viennensis* adult females were homogenized with liquid nitrogen, and total RNA was extracted using TRIzol reagent (Reagent Catalog No. 15596026, Ambion, CA, United States) following manufacturer’s instruction. The extracted RNA was resuspended in 20 μl of nuclease-free water. The concentration of RNA was quantified using a NanoDrop NC2000. Single-stranded cDNA was synthesized from 0.5 μg of total RNA using the PrimeScript RT reagent Kit with gDNA Eraser (Perfect Real Time) (Code No. RR047A, Takara, Dalian, China) and oligo (dT)_18_ primer according to manufacturer’s recommendations. The cDNA was diluted 5× for the subsequent RT-qPCR analyses.

### Candidate Reference Genes

A total of nine *A. viennensis* HKGs were selected as the candidate reference genes, including *18S* (GenBank Accession Number: AB926293), *28S* (KU323547), *EF1A* (MN603410), β*-actin* (MN607215), *Actin3* (MN603409), *V-ATPase B* (MN603411), α*-tubulin* (MN603413), *RPS9* (MN603415), and *GAPDH* (MN603412). Primer3Plus was used to design primers for RT-qPCR analysis^1^ (Untergasser et al., 2007). The specific design setting was as below: Primer Tm Min 57°C, Opt 60°C, Max 63°C, Product Size Min 90, Opt 120, Max 150, and the rest settings were by default. PCR was performed in 50 μl reactions containing 25 μl of GoTaq Green Master Mix (Reagent Catalog No. M7122, Promega, Madison, WI, United States), 1.5 μl of each primer (10 μM each), and 50 ng of the first-strand cDNA. The PCR parameters were as follows: 94°C for 3 min; 35 cycles of 94°C for 30 s, 55°C for 1 min, and 72°C for 1 min; and a final cycle of 72°C for 10 min. PCR products were separated on agarose gels, corresponding bands were extracted and purified, and the resultant DNA fragments were cloned into the pGEM^®^ -T Easy vector (Reagent Catalog No. A1360, Promega, Madison, WI, United States) and sequenced by Personal Biotechnology Co. (Shanghai, China) for confirmation.

### Extrinsic Conditions

#### Temperature

*Amphitetranychus viennensis* was placed in three climatic chambers at 16, 26, and 36°C (16L: 8D photoperiod and 60% RH), respectively, and provisioned with peach leaves. After 6 h, 40 female adults were collected from each temperature treatment.

#### Humidity

*Amphitetranychus viennensis* feeding with peach leaves were placed in climatic chambers at 30, 50, and 70% humidity, respectively, under 26°C and 16L: 8D photoperiod. After 24 h, 40 adult females were collected from each humidity treatment.

#### Photoperiod

*Amphitetranychus viennensis* feeding with peach leaves were placed in climatic chambers at photoperiod 16L: 8D, 12L: 12D, 8L: 16D, respectively, under 26°C and 50% humidity. After 7 days, 40 adult females were collected from each photoperiod.

#### Host Plants

*Amphitetranychus viennensis* were maintained in growth chambers and provisioned with leaves from four different host plants, respectively, for 3 months, including peach, *P. davidiana*, apple, *Malus pumila*, walnut, *Juglans regia*, and cherry blossom, *Prunus lannesiana*. Growth chambers were kept at 26°C, 50% RH, and 16L: 8D photoperiod. After 3 months, 40 adult females were collected from each host plant.

#### Dietary RNAi

For dietary RNAi, *A. viennensis V-type proton ATPase catalytic subunit A* (*V-ATPase A*; MN617829) was the intended target gene following a previous study in *T. urticae* (Kwon et al., 2013). A 489 bp fragment within *A. viennensis*, which shared 88% of the sequence similarity with *T. urticae V-ATPase A* (XM_015930216) was used as the template to synthesize *A. viennensis V-ATPase A* dsRNA (Supplementary Figures S1, S2). A 496 bp fragment of green fluorescent protein (*GFP*) was used as a control. The primers used for RT-qPCR analysis and dietary RNAi were listed in Supplementary Table S1. *In vitro* synthesis of dsRNAs was carried out in a T7 RiboMAX Express RNAi System (Promega, Madison, WI, United States) following manufacturer’s instruction. All resultant dsRNAs were resuspended in the nuclease-free water and stored at −80°C.

dsRNA was delivered by peach leaf with the petiole soaking in the dsRNA solution. The 250 μl of dsRNA (500 ng/μl concentration) was placed in a 0.2 ml centrifuge tube for the first day, and 50 μl of dsRNA was added on the following days for the duration of the experiment. About 200 newly molted adult females were placed on the peach leaf for 6 days. Dietary RNAi bioassay was maintained at 26°C, 50% RH, and 16L: 8D photoperiod. The GFP dsRNA was used as an exogenous control to account for the unintended silencing effects, and H_2_O is the vehicle control. A total of 40 *A. viennensis* were collected after for each treatment 6 days.

For all tested extrinsic conditions, a total of three biological replications were sampled for each treatment. Samples were snap frozen immediately in liquid nitrogen before storage at −80°C.

### Real-Time Quantitative PCR Analysis

Real-time quantitative reverse transcription PCR reactions were carried out in a CFX96 Real-Time PCR Detection System (Bio-Rad). The 20 μl reaction system contained 6.8 μl of ddH_2_O, 10 μl of 2× TB Green Premix Taq II (Tli RNaseH Plus) (Code No. RR820A, Takara, Dalian, China), 0.8 μl of each specific primer (10 μM), and 1.6 μl of first-strand cDNA template. RT-qPCR program were as follows 95°C for 3 min, 40 cycles of 95°C for 5 s, 55°C for 30 s, and 72°C for 30 s, followed by melt curve analysis using default parameters by steady increase in temperature from 55 to 90°C. Three technical replicates were carried out for each biological replicate. The existence of a single peak in the melting curve analysis was used to confirm gene-specific amplification and to rule out the non-specific amplification and the generation of primer-dimer. The standard curve for each candidate was generated from cDNA 10-fold dilution series (1/10, 1/10^2^, 1/10^3^, 1/10^4^, and 1/10^5^). The corresponding RT-qPCR efficiencies (E) were calculated following the equation: *E* = (10^[–1/slope]^ − 1) × 100%.

### Stability Analysis of Candidate Reference Genes

Four algorisms, geNorm (Vandesompele et al., 2002), NormFinder (Andersen et al., 2004), BestKeeper (Pfaffl et al., 2004), and ΔCt method (Silver et al., 2006), were used to evaluate the stability of candidate reference genes. According to geNorm, stability is assessed by the M-value, and a gene with the lowest M-value is the most stable one. More importantly, geNorm performs pairwise comparisons of one selected gene to others, and calculates a serial value of Vn/Vn + 1. When Vn/Vn + 1 > 0.15, it indicates that an additional reference gene could be added to improve normalization. *Vise verse*, when Vn/Vn + 1 < 0.15, it means that n is the optimal number of reference genes, and the inclusion of an additional reference gene (n + 1) is not needed. NormFinder calculates the stability value (SV) for each reference gene, while BestKeeper, rank the stability based on the standard deviation (SD) and percentage covariance (CV) of average cycle threshold (Ct) values. ΔCt method determines the stability of candidate reference genes through pairwise comparisons. Using raw Ct value, the average SD of each gene set is inversely proportional to its stability.

Finally, RefFinder, a web-based tool integrating all four major computational algorisms (geNorm, NormFinder, BestKeeper, and ΔCt method), provided the overall ranking of the candidates through the geometric mean of the attributed weights of each algorism (Xie et al., 2012).

### Validation of the Recommended Reference Genes

The reliability of the recommended reference genes was evaluated using a target gene, *V-ATPase A*, and a dietary RNAi bioassay. As the target gene, *A. viennensis V-ATPase A* expression was significantly reduced after the ingestion of *V-ATPase A* dsRNA, in comparison to both H_2_O and GFP controls. The relative expression of *A. viennensis V-ATPase A* was calculated using the 2^–ΔΔ*Ct*^ method (Livak and Schmittgen, 2001), and normalized to the recommended reference genes. A total of six normalization factors (NFs) were examined, including (1) the most suited and the least suited gene, (2) the top two most suited and least suited genes, and (3) the top three most suited genes and least suited genes (as recommended by RefFinder). One-way ANOVA was used to compare the gene expression level under each treatments.

## Results

### Primer Specificity and Cycle Threshold of the Candidate Reference Genes

For each reference gene, primers specificity was confirmed by a single RT-qPCR amplicon with the expected size on agarose gel and a single peak in the melting curve analysis. The PCR amplification efficiencies (E) for all the primer sets were calculated using a 10-fold dilution series of pooled cDNAs. The correlation coefficient (*R*^2^) and E for each standard curve were shown in Supplementary Table S1. The *R*^2^ ranged between 0.98 and 1.00, and efficiency of RT-qPCR ranged between 90 and 110% (Supplementary Table S1). These results suggest that all primer sets met the standard requirement for RT-qPCR analyses (Taylor et al., 2010).

**FIGURE 1:**
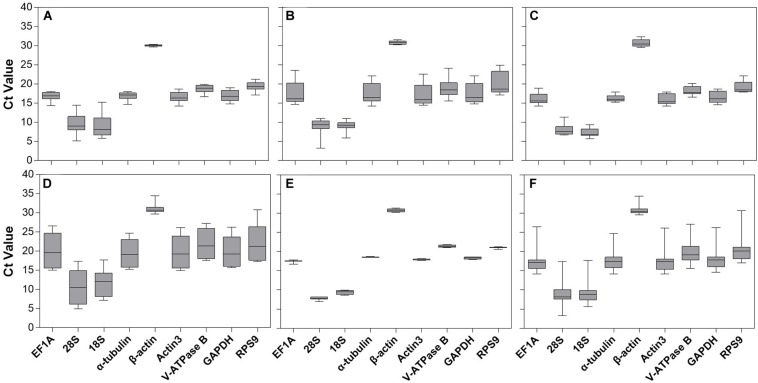
Expression range of Ct values of nine candidate reference genes under different experimental conditions in *A. viennensi*. The extrinsic conditions including temperature **(A)**, humidity **(B)**, photoperiod **(C)**, host plant **(D)**, dietary RNAi **(E)**, and all the selected extrinsic conditions **(F)**. The box indicates the 25th and 75th percentiles, the line across the box represents the median, and whisker caps the maximum and minimum values.

Gene expression analyses of the nine candidate reference genes exhibited a broad range of Ct values, covering all the experimental conditions (Figure 1). The mean Ct values ranged from 9.07 to 30.67, and they predominantly distributed between 15 and 20 (Figure 1 and Table 1). 28S and 18S rRNA were the most abundant transcripts with the mean Ct values of 9.07 and 9.40, while β*-actin* was the least (mean Ct value = 30.67, Table 1). *EF1A* had the most variable expression level, which was reflected in the highest SE value (SE = 0.44, Table 1). Ct values of the candidates varied significantly across different experimental conditions. Host plant was the most effective contributor for such variation. Among them, Ct values from apple and peach were lower than cherry blossom and walnut, while most of the SE values from different host plants were above 1.0 (Figure 1 and Table 1, Ct values under different host plants were shown in Supplementary Table S2).

**TABLE 1:** Ct values of candidate reference genes under different extrinsic conditions.

**Candidate genes**	**Temperature (Ct ± SE)**	**Humidity (Ct ± SE)**	**Photoperiod (Ct ± SE)**	**Host plant (Ct ± SE)**	**Dietary RNAi (Ct ± SE)**	**Extrinsic conditions (Ct ± SE)**
*EF1A*	16.780.38	17.691.08	16.070.52	20.001.33	17.490.10	17.750.44
*28S*	9.480.88	8.910.77	8.040.50	10.591.29	7.830.13	9.070.42
*18S*	8.980.98	9.030.46	7.200.40	11.681.06	9.370.16	9.400.40
α*-tubulin*	16.830.33	17.640.92	16.180.30	19.341.09	18.580.03	17.820.36
β*-actin*	30.000.07	30.770.15	30.660.30	31.020.36	30.780.14	30.670.12
*Actin3*	16.610.44	17.290.96	15.920.45	19.821.30	17.890.05	17.650.43
*V-ATPase B*	18.740.32	18.770.84	18.160.41	21.911.18	21.390.10	19.930.41
*GAPDH*	17.010.47	17.590.91	16.500.50	19.981.19	18.330.08	18.010.40
*RPS9*	19.440.39	20.181.00	19.140.55	22.181.38	21.070.06	20.510.43

### Stability of the Candidate Reference Genes

#### Stability and Optimal Number of Reference Genes Based on geNorm

For temperature, the stability from high to low was *EF1A* = α*-tubulin*, *V-ATPase B*, *RPS9*, *Actin3*, *GAPDH*, β*-actin*, *28S*, and *18S* (Table 2). For humidity, three of the most stable candidates were *GAPDH* = *RPS9*, and *Actin3* (Table 2). For photoperiod, the top three most stable reference genes were *RPS9* = *EF1A*, and *Actin3* (Table 2). In the host plant study, geNorm ranked the top three most stables reference genes as *Actin3* = *GAPDH*, *V-ATPase B* (Table 2). For dietary RNAi, the stability from high to low was *GAPDH* = *V-ATPase B*, *RPS9*, α*-tubulin*, *Actin3*, *EF1A*, *28S*, *18S*, and β*-actin* (Table 2). Under all the extrinsic conditions, the top three most stable candidate genes were *GAPDH* = *Actin3*, and *EF1A* (Table 2).

**TABLE 2:** Stability of candidate reference genes under different extrinsic conditions.

**Conditions**	**Candidate genes**	**geNorm**		**NormFinder**		**BestKeeper**		**Delta Ct**		**RefFinder recommendation**
						
		**Stability**	**Ranking**	**Stability**	**Ranking**	**Stability**	**Ranking**	**Stability**	**Ranking**	
Temperature	*EF1A*	0.259	1	0.548	3	0.911	4	1	1	*EF1A* α*-tubulin Actin3*
	*28S*	1.054	8	1.95	8	2.012	8	2.18	8	
	*18S*	1.396	9	2.491	9	2.325	9	2.593	9	
	α*-tubulin*	0.259	1	0.655	5	0.793	2	1.042	4	
	β*-actin*	0.627	7	1.311	7	0.173	1	1.578	7	
	*Actin3*	0.381	5	0.141	1	1.113	6	1.014	2	
	*V-ATPase B*	0.277	3	0.6	4	0.815	3	1.039	3	
	*GAPDH*	0.431	6	0.149	2	1.236	7	1.055	5	
	*RPS9*	0.313	4	0.659	6	0.936	5	1.063	6	
Humidity	*EF1A*	0.691	4	2.259	7	2.43	8	2.606	5	*GAPDH 18S Actin3*
	*28S*	2.595	9	3.101	9	1.517	3	3.443	9	
	*18S*	1.355	5	1.026	1	0.939	2	2.3	4	
	α*-tubulin*	2.353	8	2.536	8	2.239	6	3.103	8	
	β*-actin*	1.769	6	1.726	4	0.366	1	2.623	6	
	*Actin3*	0.497	3	1.538	3	2.314	7	2.194	2	
	*V-ATPase B*	2.061	7	1.776	6	1.864	4	2.668	7	
	*GAPDH*	0.440	1	1.448	2	2.179	5	2.128	1	
	*RPS9*	0.440	1	1.774	5	2.493	9	2.294	3	
Photoperiod	*EF1A*	0.601	1	0.829	6	1.18	7	1.109	6	*V-ATPase B Actin3 18S*
	*28S*	0.931	6	0.749	4	1.086	5	1.104	5	
	*18S*	0.868	5	0.667	3	0.956	4	1.057	3	
	α*-tubulin*	1.025	8	0.856	8	0.672	1	1.174	8	
	β*-actin*	1.128	9	1.312	9	0.74	2	1.489	9	
	*Actin3*	0.745	3	0.659	2	1.117	6	1.029	2	
	*V-ATPase B*	0.782	4	0.419	1	0.949	3	0.936	1	
	*GAPDH*	0.977	7	0.838	7	1.204	8	1.157	7	
	*RPS9*	0.601	1	0.809	5	1.238	9	1.096	4	
Host plant	*EF1A*	0.785	5	0.954	5	4.306	9	1.594	5	*GAPDH V-ATPase B* α*-tubulin*
	*28S*	0.98	6	1.296	6	4.053	6	1.856	6	
	*18S*	1.187	7	1.51	7	3.196	2	2.085	7	
	α*-tubulin*	0.723	4	0.270	1	3.508	3	1.465	4	
	β*-actin*	1.927	9	3.762	9	0.816	1	3.89	9	
	*Actin3*	0.538	1	0.706	4	4.21	7	1.457	3	
	*V-ATPase B*	0.598	3	0.270	1	3.84	5	1.352	1	
	*GAPDH*	0.538	1	0.372	3	3.825	4	1.425	2	
	*RPS9*	1.365	8	1.844	8	4.279	8	2.215	8	
Dietary RNAi	*EF1A*	0.257	6	0.354	8	0.196	4	0.417	8	*GAPDH V-ATPase B RPS9*
	*28S*	0.294	7	0.285	6	0.271	7	0.383	6	
	*18S*	0.318	8	0.333	7	0.401	9	0.406	7	
	α*-tubulin*	0.199	4	0.188	4	0.067	1	0.308	4	
	β*-actin*	0.354	9	0.424	9	0.345	8	0.46	9	
	*Actin3*	0.217	5	0.257	5	0.139	3	0.346	5	
	*V-ATPase B*	0.085	1	0.092	2	0.234	6	0.278	2	
	*GAPDH*	0.085	1	0.062	1	0.205	5	0.270	1	
	*RPS9*	0.168	3	0.153	3	0.131	2	0.300	3	
Extrinsic conditions	*EF1A*	0.758	3	1.182	5	2.166	6	1.714	3	*GAPDH Actin3 EF1A*
	*28S*	1.677	8	1.915	8	2.17	7	2.31	8	
	*18S*	1.490	7	1.448	7	1.981	3	1.995	7	
	α*-tubulin*	1.313	6	1.161	4	1.947	2	1.828	6	
	β*-actin*	1.897	9	2.362	9	0.591	1	2.664	9	
	*Actin3*	0.609	1	0.811	2	2.132	5	1.525	2	
	*V-ATPase B*	1.134	5	1.099	3	2.229	8	1.762	5	
	*GAPDH*	0.609	1	0.703	1	2.042	4	1.514	1	
	*RPS9*	0.876	4	1.235	6	2.256	9	1.759	4	

**FIGURE 2:**
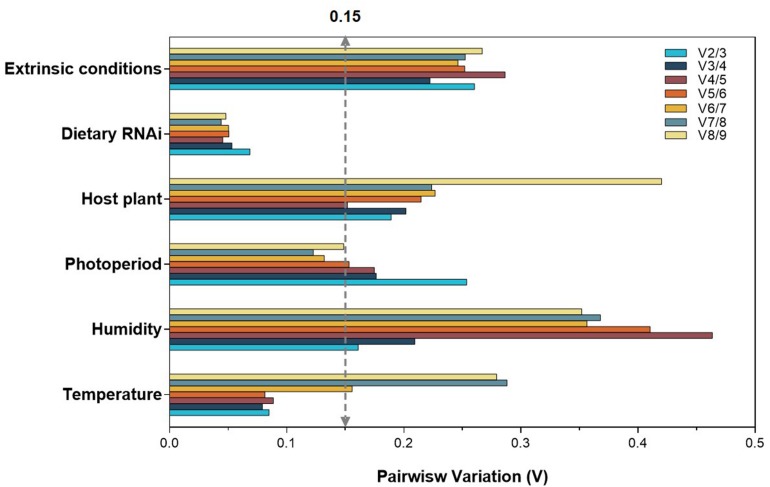
Optimal number of reference genes for the normalization of *A. viennensi* under selected extrinsic experimental conditions. The pairwise variation (Vn/Vn + 1) was analyzed for the normalization factors by geNorm program to determine the optimal number of reference genes included in the RT-qPCR analysis. Values less than 0.15 indicate that another reference gene will not significantly improve normalization.

To produce accurate and consistent results, multiple rather than a single normalizer is highly encouraged. The optimal number of reference genes is determined by a V-value (Vn/n + 1) from geNorm. The cutoff value of 0.15 indicates that an additional reference gene (Vn + 1) is not necessary for the reliable normalization, i.e., *n* is the optimal number. In the case of temperature and dietary RNAi, V2/3 was less than 0.15, suggesting that two reference genes are sufficient for normalization (Figure 2). For photoperiod, the first V-value <0.15 appeared at V6/7, suggesting that six reference genes are necessary for the reliable normalization (Figure 2). Although all the V-values were higher than 0.15, the lowest was at V2/3 for humidity, suggesting that two reference genes are the optimal number (Figure 2). For the same reason, the lowest V-values was at V4/5, implying that four reference genes are necessary for normalization among host plants (Figure 2).

#### Stability of the Candidate Reference Genes Based on NormFinder

For temperature, NormFinder ranked the stability from high to low as: *Actin3*, *GAPDH*, *EF1A*, *V-ATPase B*, α*-tubulin*, *RPS9*, β*-actin*, *28S*, and *18S* (Table 2). For humidity, the stability from high to low was *18S*, *GAPDH*, *Actin3*, β*-actin*, *RPS9*, *V-ATPase B*, *EF1A*, α*-tubulin*, and *28S* (Table 2). For photoperiod and host plants, the top three most stable reference genes were *V-ATPase B*, *Actin3*, and *18S;* and *V-ATPase B* = α*-tubulin*, and *GAPDH*, respectively (Table 2). For dietary RNAi, the top three most stables reference genes were *GAPDH, V-ATPase B*, and *RPS9* (Table 2). Under all the extrinsic conditions, the top three most stable candidate genes were *GAPDH*, *Actin3*, and *V-ATPase B* (Table 2).

#### Stability of the Candidate Reference Genes Based on BestKeeper

The candidate with the lowest SD is considered as the most stable reference gene. In this study, β*-actin*, α*-tubulin*, and *V-ATPase B* were the most stably expressed candidates under different temperature (Table 2). For humidity, the stability from high to low was β*-actin*, *18S*, *28S*, *V-ATPase B*, *GAPDH*, α*-tubulin*, *Actin3*, *EF1A*, and *RPS9* (Table 2). For photoperiod, the top three most stable reference genes were α*-tubulin*, β*-actin*, and *V-ATPase B* (Table 2). For host plants, the top three most stable candidates were β*-actin*, *18S*, and α*-tubulin* (Table 2). For dietary RNAi, the top three most stable reference genes were α*-tubulin*, *RPS9*, *Actin3*. In summary, the stability of reference genes from high to low was β*-actin*, α*-tubulin*, *18S*, *GAPDH*, *Actin3*, *EF1A*, *28S*, *V-ATPase B*, and *RPS9* combined all the extrinsic conditions (Table 2).

#### Stability of the Candidate Reference Genes Based on Delta Ct

According to Delta Ct method, under different temperature, *EF1A* was the most reliable candidate (Table 2). The overall ranking from the most to the least stable candidates was *EF1A*, *Actin3*, *V-ATPase B*, α*-tubulin*, *GAPDH*, *RPS9*, β*-actin*, *28S*, and *18S* (Table 2). For humidity, *GAPDH*, *Actin3*, and *RPS9* were ranked as the top three most stable reference genes (Table 2). For photoperiod, the top-ranked candidate gene was *V-ATPase B* (Table 2). The stability from high to low was *V-ATPase B*, *Actin3*, *18S*, *RPS9*, *28S*, *EF1A*, *GAPDH*, α*-tubulin*, and β*-actin* (Table 2). For host plants, the top three most stable reference genes were *V-ATPase B*, *GAPDH*, and *Actin3* (Table 2). For dietary RNAi, the stability ranking from high to low was *GAPDH*, *V-ATPase B*, *RPS9*, α*-tubulin*, *Actin3*, *28S*, *18S*, *EF1A*, and β*-actin* (Table 2). Combining all the extrinsic conditions, *GAPDH* and *Actin3* were ranked as the top candidates (Table 2). The overall ranking of the most to the least stable candidates was *GAPDH*, *Actin3*, *EF1A*, *RPS9*, *V-ATPase B*, α*-tubulin*, *18S*, *28S*, and β*-actin* (Table 2).

### Recommended Reference Genes by RefFinder

**FIGURE 3:**
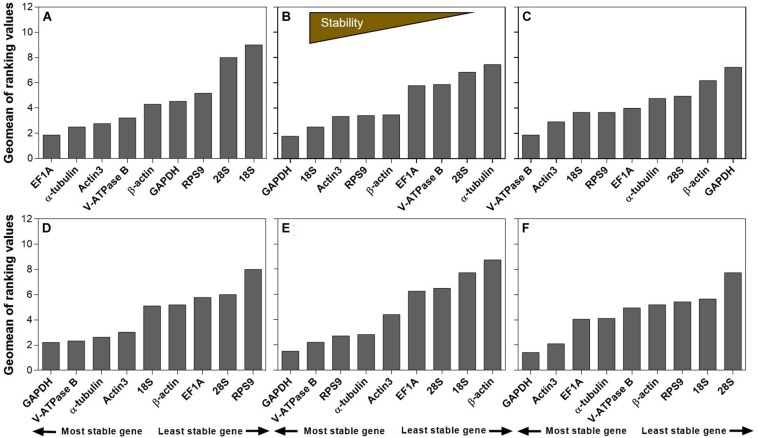
Expression stability of candidate reference genes under different experimental conditions in *A. viennensi*. The extrinsic conditions including temperature **(A)**, humidity **(B)**, photoperiod **(C)**, host plant **(D)**, dietary RNAi **(E)**, and all the selected extrinsic conditions **(F)**. The final stability ranking was provided by ReFinder, which is a comprehensive platform integrating all four algorithms used in this study. A lower Geomean value suggests stable expression.

The stability ranking of the nine candidates under all the extrinsic conditions was summarized in Table 2. RefFinder is a web-based software, which provides a comprehensive stability ranking by integrating the inputs from all four major algorithms, including geNorm, NormFinder, BestKeeper, and the comparative ΔCt method. Based on the individual ranking, RefFinder assigns an appropriate weight to an individual gene and calculated the geometric mean of their weights for the overall final ranking. For temperature, *EF1A*, α*-tubulin*, and *Actin3* were the most stable candidates (Figure 3). For humidity, *GAPDH*, *18S*, and *Actin3* were the top three candidates (Figure 3). For photoperiod, the ranking from the most to the least stable candidates was *V-ATPase B*, *Actin3*, *18S*, *RPS9*, *EF1A*, α*-tubulin*, *28S*, β*-actin*, and *GAPDH* (Figure 3). For host plants, *GAPDH* was the most stably expressed gene, the remaining ranking was *V-ATPase B*, α*-tubulin*, *Actin3*, *18S*, β*-actin*, *EF1A*, *28S*, and *RPS9* (Figure 3). For dietary RNAi, *GAPDH*, *V-ATPase B*, and *RPS9* were the most stable candidates (Figure 3). Combining all the extrinsic conditions, the order of stability from high to low was *GAPDH*, *Actin3*, *EF1A*, α*-tubulin*, *V-ATPase B*, β*-actin*, *RPS9*, *18S*, and *28S* (Figure 3).

### Validation of Recommended Reference Genes

**FIGURE 4:**
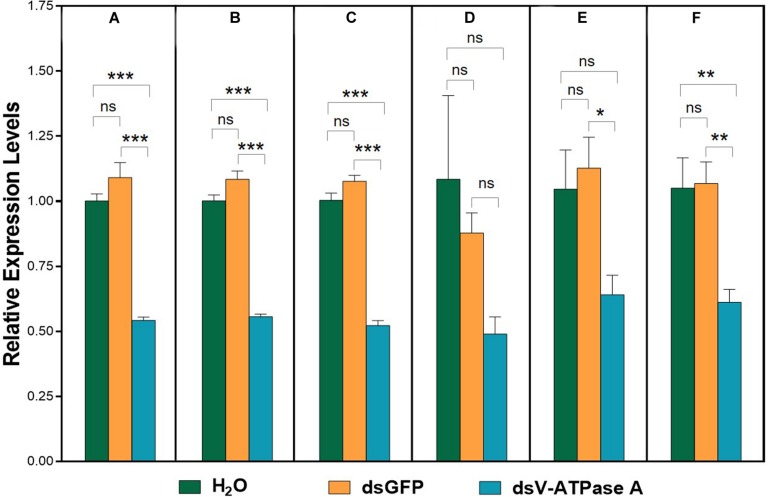
Validation of the recommended reference gene(s). Expression profiles of *V-ATPase A* under dsRNA treatments were investigated using six normalization factors. *V-ATPase A* expression levels were analyzed when normalization for the most suited (*GAPDH*) **(A)**, the top two most suited (*GAPDH*, *V-ATPase B*) **(B)**, the top three most suited (*GAPDH*, *V-ATPase B*, *RPS9*) **(C)**, the least suited (β*-actin*) **(D)**, and the top two least suited (β*-actin*, *18S*) **(E)**, and the top three least suited reference genes (β*-actin*, *18S*, *28S*) **(F)**. Bars represent the means standard error of three biological replicates. Asterisk indicate significant differences between the treatments and controls (***P < 0.001, **P < 0.01, *P < 0.05, ns indicate no significant difference).

For dietary RNAi bioassay, *A. viennensis V-ATPase A* expression was significantly suppressed in comparison to both H_2_O and GFP controls when its expression was normalized to all six normalization factors (NFs), except the least suited gene, β*-actin*, and the top two least suited genes, β*-actin* and *18S* (Figure 4). The expression patterns of *A. viennensis V-ATPase A* were similar using the most stable reference gene (*GAPDH*, Figure 4A), the top two most suited reference genes (*GAPDH* and *V-ATPase B*, Figure 4B), and the top three most suited reference genes (*GAPDH*, *V-ATPase B*, and *RPS9*, Figure 4C). Although *A. viennensis V-ATPase A* expression was numerically suppressed using the least suited reference gene (β*-actin*), however, the expression level was not significantly different between dsV-ATPase A treatment and dsGFP and H_2_O controls (Figure 4D). Similarly, *V-ATPase A* expression was not significantly different between dsV-ATPase A treatment and H_2_O control when the top two least suited reference genes (β*-actin* and *18S*) were used as the normalizer (Figure 4E). The significance was restored when we used the top three least suited reference genes (β*-actin*, *18S*, and *28S*) as the normalizer (Figure 4F), which was consistent with the results from suited reference gene(s).

## Discussion

Real-time quantitative reverse transcription PCR is a powerful tool to analyze gene expression. However, the reliability and accuracy of RT-qPCR analysis are often affected by the quality of RNA and the efficiency of reverse transcription. Consequently, the selection of appropriate reference genes is critical in offsetting variations and biases (Lü et al., 2018). Recently, there has been a dramatic increase in studies of reference gene selection in the cell-content feeding arthropods, including Hemiptera (Supplementary Table S3; Li et al., 2013; Yang et al., 2014, 2015b; Yuan et al., 2014; Shang et al., 2015; Koramutla et al., 2016; Ma et al., 2016; An et al., 2016; Yu et al., 2016; Arya et al., 2017; Kang et al., 2017), and three spider mites species (Sun et al., 2010; Niu et al., 2012; Yue et al., 2013; Yang et al., 2015a). Most of these studies in Tetranychidae species have focused on the expression patterns of reference genes under the impact of insecticide and developmental stage. In Niu et al. (2012), the mRNA expression profiles of seven HKGs were analyzed under different stress treatments in *P. citri*, including thermos, UV irradiation, and acid rain. Most recently, we searched for the internal references from a pool of *A. viennensis* HKGs under diverse intrinsic conditions, including developmental stage, sex, and diapause (Yang et al., 2019). Here, we continued our efforts to include reference genes for an array of extrinsic conditions, including temperature, humidity, photoperiod, host plant, and dietary RNAi.

In this study, host plants caused the most variations for the Ct values in *A. viennensis*. Similar studies in other arthropods, however, have not found such dramatically variations in Ct values (the cotton mealybug, *P. solenopsis*, Arya et al., 2017; and the green peach aphid, *M. persicae*, Kang et al., 2017). Our results suggest that host plants can cause significant changes on arthropods at the molecular level. Under the same rearing conditions, *A. viennensis* feeding on different host plants had a significant impact on their life history traits, including developmental time, female lifespan, oviposition period, and fecundity (Qiao, 2001). In this study, variations of Ct values in apple and peach were lower than cherry blossom and walnut.

**FIGURE 5:**
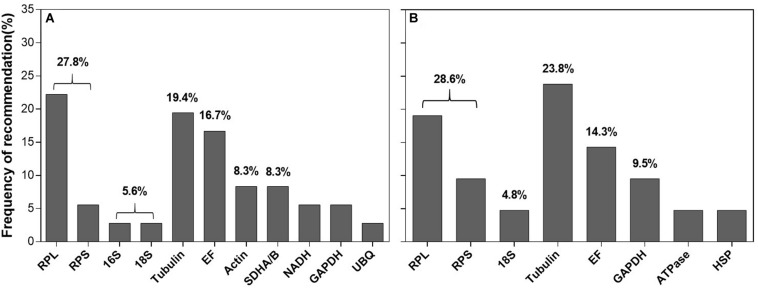
Frequency of reference genes recommended for the cell-content feeding arthropods. Here we surveyed the frequency of each reference gene recommended under different temperature among the twelve cell-content feeding species **(A)** and different host plants (or diet) among seven cell-content feeding species **(B)**. Reference genes (top three) recommended for each species under different temperature and host plant are detailed in Supplementary Table S3.

Within the reference gene selection studies, temperature and host plants (or diet) are the most extensively investigated extrinsic conditions among the cell-content feeding arthropods. Figure 5 and Supplementary Table S3 summarized the top three recommended reference genes under these two extrinsic conditions in different cell-content feeders. For temperature, based on frequency of being recommended, ribosomal proteins and rRNAs were accounted for 33.4% (27.8% for ribosomal proteins + 5.6% for rRNA) (Figure 5A). For host plants, the trend was the same (28.6% for ribosomal proteins + 4.8% for rRNA = 33.4%, Figure 5B). Ribosomal proteins and rRNAs were also the common choice for the intrinsic conditions (specifically, developmental stages) among the cell-content feeding arthropods (Yang et al., 2019). Because of their abundance in the cell, ribosomal proteins and rRNAs are consistently stably expressed across diverse conditions. Not surprisingly, they have been adopted by many as the internal references (Lü et al., 2018; Yang et al., 2018, 2019). However, the subtle changes of the target gene expression potentially can be overshadowed by the dominance of rRNAs in the total RNAs (>80%), which affects the utility of ribosomal proteins and rRNAs for the normalization (Yang et al., 2018). That been said, ribosomal proteins and rRNAs are still employable if their abundance is comparable to the target gene(s), i.e., Ct values are similar.

Previous studies suggest that there is no “universal” reference gene that is applicable across all the experimental conditions (Lü et al., 2018). Based on our previous and current studies, few reference genes, however, are consistently stably expressed throughout intrinsic and extrinsic conditions, respectively, in *A. viennensis* (Table 3). As a whole, the same three reference genes were recommended for the three intrinsic conditions, including developmental stage, sex, and diapause (Yang et al., 2019), while the number of recommended genes increased to eight for the five extrinsic conditions, accounting for temperature, humidity, photoperiod, host plant, and dietary RNAi. It is not a surprise to see more variations in recommended reference genes under extrinsic than intrinsic conditions. Overall, *V-ATPase B*, *GAPDH*, and *Actin3* were the most commonly selected reference genes in *A. viennensis* regardless of the experimental conditions.

**TABLE 3:** Recommended reference genes under different intrinsic and extrinsic conditions.

**Conditions**	**Recommendation**
**Intrinsic**	
Developmental stage	*V-ATPase B, Actin3*, *GAPDH*
Sex	*Actin3*, *GAPDH*, *V-ATPase B*
Diapause	*Actin3, GAPDH, V-ATPase B*
**Extrinsic**	
Temperature	*EF1A*, α*-tubulin*, *Actin3*
Humidity	*GAPDH, 18S, Actin3*
Photoperiod	*V-ATPase B, Actin3, 18S*
Host plant	*GAPDH, V-ATPase B*,α*-tubulin*
Dietary RNAi	*GAPDH, V-ATPase B, RPS9*

In recent years, a single reference gene has gradually been replaced with the multiple normalizer for RT-qPCR analysis (Lü et al., 2018; Yang et al., 2018). Results from dietary RNAi bioassay justified this movement. When we used the most suited/stable candidate genes as the internal reference(s), *V-ATPase A* expression was significantly suppressed after *A. viennensis* ingested *V-ATPase A* dsRNA. The significance, however, was compromised when *V-ATPase A* expression was normalized to the least suited reference gene (β*-actin*), while such significance was restored when the top three least suited reference genes (β*-actin*, *18S*, and *28S*) were used as the normalizer.

## Data Availability Statement

The datasets generated for this study can be found in the GenBank accession numbers: MN603410, MN607215, MN603409, MN603411, MN603413, MN603415, and MN603412.

## Author Contributions

XZ, RF, and JY designed the experiments. ZL and YG collected the samples from field. JY, YZ, and JZ carried out the experiments and did the analysis. JY drafted the manuscript. XZ revised the manuscript. All authors have read and approved its final version.

## Conflict of Interest

The authors declare that the research was conducted in the absence of any commercial or financial relationships that could be construed as a potential conflict of interest.

## Footnotes

1 http://www.bioinformatics.nl/cgi-bin/primer3plus/primer3plus.cgi
