# Supplementary material for: Selection of Reference Genes for RT-qPCR Analysis Under Extrinsic Conditions in the Hawthorn Spider Mite, Amphitetranychus viennensis
Source: Front Physiol. 2020 Apr 21;11:378. doi: 10.3389/fphys.2020.00378 (PMC7187807; doi:10.3389/fphys.2020.00378)
Supplement: Supplementary file 1 [file Table_1.DOCX]

**SUPPLEMENTARY MATERIALS**

**
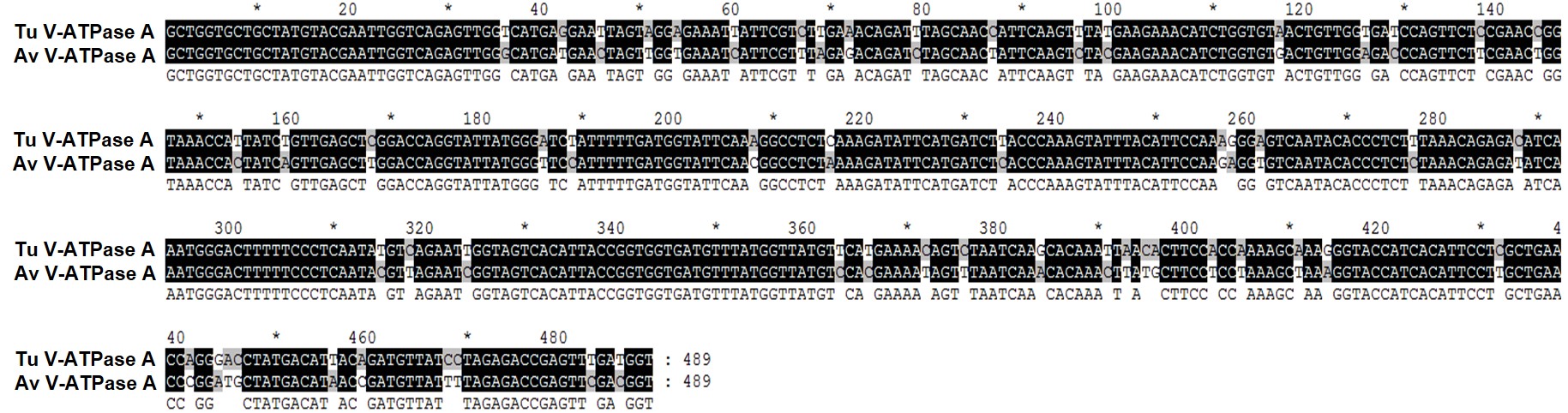
**

**Figure S1. The alignment of a highly conserved region within the *V*-*ATPase A* from *T. urticae* and *A. viennensis* (Tu indicated *T. urticae*; Av indicated *A. viennensis*).** This 489bp fragment was selected as the target template to synthesis dsRNAs. *V-ATPase A* fragment from *A. viennensis* has a sequence similarity 88% with *T. urticae*.


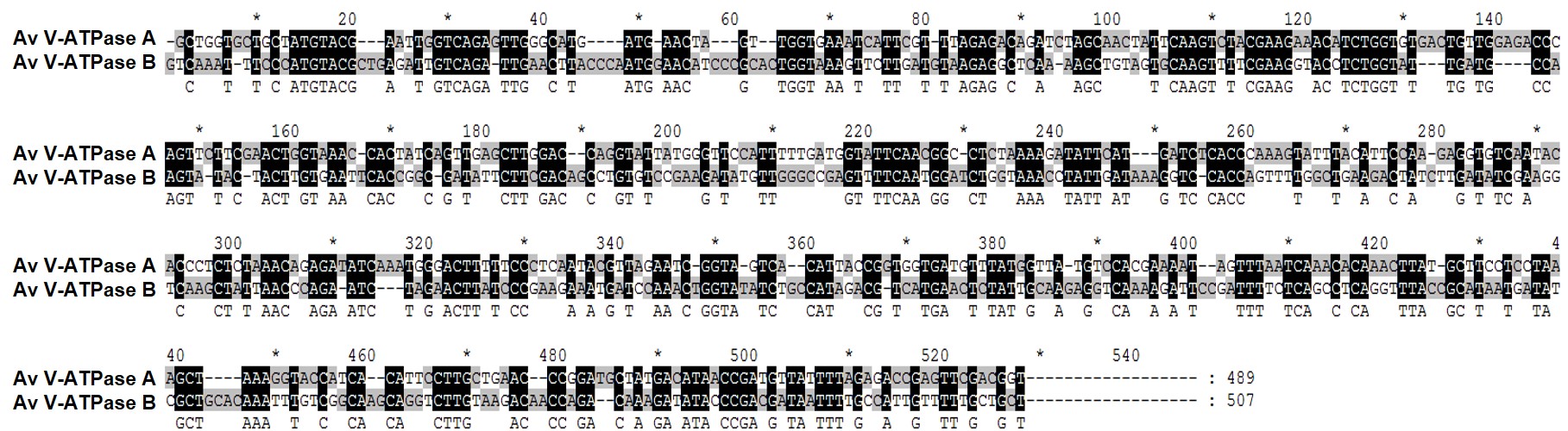


**Figure S2. The alignment of a highly conserved region within the *V*-*ATPase A* and *V*-*ATPase B* from *A. viennensis* (Av indicated *A. viennensis*).** This 489bp fragment of *V-ATPase A* was selected as the target template to synthesize dsRNAs. *V-ATPase A* and *V-ATPase B* fragment from *A. viennensis* has a 49% sequence similarity.
