# Supplementary material for: Selection of Reference Genes for RT-qPCR Analysis Under Extrinsic Conditions in the Hawthorn Spider Mite, Amphitetranychus viennensis
Source: Front Physiol. 2020 Apr 21;11:378. doi: 10.3389/fphys.2020.00378 (PMC7187807; doi:10.3389/fphys.2020.00378)
Supplement: Supplementary file 2 [file Table_2.DOCX]

**SUPPLEMENTARY MARTIALS**

**Table S1. Primers used in this study.**

| **Gene name** | **Gene symbol** | **Primers (5′-3′)** | **Amplicon length (bp)** | **Accession No.** | **Efficiency (%)** | **R^2^** |
| --- | --- | --- | --- | --- | --- | --- |
| **Primers for RT-qPCR analysis of candidate reference genes** | | | | | | |
| *18S ribosomal RNA* | *18S* | F: CCGCCCTAGTTCTAACCATAAA  R: GTTTCAGCTTTGCAACCATACT | 132 | AB926293 | 102.4 | 0.98 |
| *28S ribosomal RNA* | *28S* | F: AGCTAAGACCCCTTGGCAAC  R: TAAGGATAGGGGGCCTTCCC | 132 | KU323547 | 99.3 | 0.99 |
| *Elongation factor 1-alpha* | *EF1A* | F: AGGGTTCCAAATTGGAAGGTAAA  R: GTGGAAGTCGAAGAGCCTTGT | 93 | MN603410 | 99.1 | 1.00 |
| *β-actin* | *β-actin* | F: TGCCCCAAGAGCTGTATTCC  R: TTGGGCTTCGTCTCCAACAT | 103 | MN607215 | 103.3 | 0.995 |
| *Actin3* | *Actin3* | F: CCAAGGAGTAATGGTCGGTATG  R: CCATGCTCAATTGGGTATTTAAGG | 102 | MN603409 | 90.3 | 0.99 |
| *vacuolar-type H+-ATPase B* | *V-ATPase B* | F: GTCGTGGTTTCCCAGGTTAT  R: CGTTTGGCATGGTAAGAATGG | 117 | MN603411 | 93.6 | 0.98 |
| *α-tubulin* | *α-tubulin* | F: ATTCGTTGACTGGTGCCCAA  R: TGCCCGTTGAACTTTGGCTA | 97 | MN603413 | 95.0 | 0.98 |
| *40S ribosomal protein S9* | *RPS9* | F: CTGCTCGAGAGTTGCTGACT  R: AGACCCAAGACGTAATCGAGC | 133 | MN603415 | 106.5 | 0.99 |
| *Glyceraldehyde 3-phosphate*  *dehydrogenase* | *GAPDH* | F: TGATGCACCCATGTTTGTAAT  R: GGGGCAAGACAGTTAGTGGTA | 99 | MN603412 | 90.6 | 0.99 |
| **Primers for RT-qPCR analysis of target genes** | | | | | | |
| *ATPase catalytic subunit A* | *V-ATPase A* | F: TGGAAGTTGCCCGTCTTATT  R: GGTAGCCTCAATGGCATGTT | 149 | MN617829 | 102.1 | 1.00 |
| **Primers for dsRNA in vitro synthesis** | | | | | | |
| *ATPase catalytic subunit A* | *V-ATPase A* | F: GCTGGTGCTGCTATGTACGA  R: ACCGTCGAACTCGGTCTCTA | 489 | MN617829 |  |  |
| *Green fluorescent protein* | *GFP* | F: GACGTAAACGGCCACAAGTT  R: TGTTCTGCTGGTAGTGGTCG | 496 |  |  |  |

**Table S2. Ct values of nine candidate reference genes under different host plants.**

| Host plants | *EF1A* | *28S* | *18S* | *Tubulin* | *β-actin* | *Actin3* | *V-ATPase B* | *GAPDH* | *RPS9* |
| --- | --- | --- | --- | --- | --- | --- | --- | --- | --- |
| Peach1 | 15.38 | 5.24 | 7.64 | 15.74 | 30.61 | 15.80 | 18.31 | 16.031 | 17.82 |
| Peach2 | 15.00 | 8.19 | 8.64 | 15.173 | 30.31 | 15.53 | 17.59 | 16.03 | 17.52 |
| Peach3 | 15.57 | 4.89 | 7.91 | 15.53 | 29.64 | 15.08 | 17.53 | 15.69 | 17.56 |
| Apple1 | 15.48 | 5.68 | 7.16 | 15.83 | 30.46 | 14.91 | 17.91 | 16.03 | 17.27 |
| Apple2 | 16.85 | 7.57 | 10.83 | 16.87 | 31.22 | 16.10 | 18.37 | 16.63 | 18.62 |
| Apple3 | 15.86 | 7.65 | 8.71 | 15.85 | 31.41 | 16.22 | 18.71 | 16.50 | 18.59 |
| Cherry blossom1 | 22.23 | 13.56 | 14.40 | 21.40 | 30.32 | 22.37 | 24.01 | 21.94 | 23.74 |
| Cherry blossom2 | 23.46 | 15.176 | 16.85 | 22.14 | 30.25 | 23.65 | 25.23 | 23.54 | 24.70 |
| Cherry blossom3 | 24.87 | 17.36 | 17.7 | 23.31 | 31.31 | 26.11 | 26.88 | 26.25 | 25.60 |
| Walnut1 | 26.53 | 15.14 | 13.75 | 24.67 | 34.49 | 24.47 | 27.19 | 23.79 | 27.33 |
| Walnut2 | 23.85 | 13.95 | 13.31 | 22.16 | 31.57 | 23.61 | 25.07 | 22.98 | 26.61 |
| Walnut3 | 24.86 | 12.68 | 13.25 | 23.41 | 30.67 | 23.97 | 26.12 | 24.32 | 30.75 |
| Mean | 20.00 | 10.59 | 11.68 | 19.34 | 31.02 | 19.82 | 21.91 | 19.98 | 22.18 |
| SD | 4.62 | 4.480 | 3.69 | 3.77 | 1.23 | 4.49 | 4.10 | 4.12 | 4.78 |
| SE | 1.34 | 1.29 | 1.06 | 1.09 | 0.36 | 1.30 | 1.18 | 1.19 | 1.38 |

**Table S3. Recommended reference genes for RT-qPCR analysis in cell-content feeding arthropods**

| **Species** | **Candidate genes** | **Extrinsic conditions** | | | | **Reference** | |
| --- | --- | --- | --- | --- | --- | --- | --- |
|  |  | **Temperature** | | **Host Plant /Diet** | | |  |
| **Tetranychidae** | | | | | | | |
| *Amphitetranychus viennensis* | *18S*, *28S*, *EF1A*, *β-actin, Actin3*, *V-ATPase B*, α*-tubulin*, *RPS9*, *GAPDH* | | *EF1A*, *α-tubulin*, *Actin3* | | *GAPDH*, *V-ATPase B*, *α-tubulin* | | This study |
| **Hemiptera** | | | | | | | |
| *Myzus persicae* | *Actin*, *RPL27*, *RPL7*, *β-tubulin*, *GAPDH*, *ACE*, *18S*, *EF-1A*, *RPL32* | | *Actin, 18S, RPL7* | | *EF1A, β-tubulin, RPL32* | | Kang et al., 2017 |
| *Bemisia tabaci* | *Actin, 18S, HSP20, HSP40, HSP70, HSP90, γ-tubulin, RPL29, SDHA, GAPDH, EF-1A, PPIA, NADH, Myosin L, ATPase* | | *EF1A, NADH, SDHA* | | *HSP90, RPL29, EF1A* | | Li et al., 2013 |
| *Aphis gossypii* | *18S*, *28S*, *Actin*, *GAPDH*, *EF1A*, *RPL7*, α*-tubulin*, *TBP* | | *GAPDH, RPL7 EF1A* | | 18S, *Actin,* *EF1A* | | Ma et al., 2016 |
| *Lipaphis erysimi* | *16S*, *SDHB*, *Actin*, *EF1A*, *RPL13*, *RPS18*, *RPL27*, *RPL29*, *β-tubulin*, *GAPDH* | | *RPL13, RPL27, EF1A* | | *RPL13, Actin, β-tubulin* | | Koramutla et al., 2016 |
| *Phenacoccus solenopsis* | *Actin, RPL32, β-tubulin, α-tubulin, GAPDH, SDHA* | | *RPL32, α-tubulin, SDHA* | | *GAPDH, RPL32, α-tubulin* | | Arya et al., 2017 |
| *Nilaparvata lugens* | *18S, Actin 1, Muscle actin, RPS11, RPS15, α-tubulin, EF11, ArgK* | | *RPS15, α-tubulin, EF11,* | | *RPS15, α-tubulin, RPS11* | | Yuan et al., 2014 |
| *Acyrthosiphon pisum* | *18S, 28S, 16S, Actin, EF1A, TATA, RPL12, β-tubulin, NADH, V-ATPase A, SDHB* | | *SDHB,16S, NADH,* | | NA | | Yang et al., 2014 |
| *Aphis craccivora* | *18S, 12S, EF1A, RPL11, V-ATPase D, RPL14, RPS8, RPS23, NADH, HSP70* | | *RPS8, RPL14, RPL11* | | NA | | Yang et al., 2015b |
| *Toxoptera citricida* | *18S, Actin, EF1A, GAPDH, α-tubulin, β-tubulin, RNAP II* | | *Actin, β-tubulin, EF1A* | | NA | | Shang et al., 2015 |
| *Sogatella furcifera* | *18S, Actin, EF1A, α-tubulin, GAPDH, UBQ, RPS18, RPL9, RPL10* | | *α-tubulin, RPL9, GAPDH,* | | NA | | An et al., 2016 |
| *Ericerus pela* | *Actin1, Actin2, α-tubulin, β-tubulin1, β-tubulin2, SDHA1, SDHA2, SDHA3, RNAP II, RPL50-1, RPL50-2, RPL15, UBQ1, UBQ2, Myosin* | | *β-tubulin1, β-tubulin2, UBQ1* | | NA | | Yu et al., 2016 |
